# Radiosensitization Effect of Gold Nanoparticles in Proton Therapy

**DOI:** 10.3389/fpubh.2021.699822

**Published:** 2021-07-29

**Authors:** Charnay Cunningham, Maryna de Kock, Monique Engelbrecht, Xanthene Miles, Jacobus Slabbert, Charlot Vandevoorde

**Affiliations:** ^1^Radiation Biophysics Division, Nuclear Medicine Department, iThemba LABS, National Research Foundation, Cape Town, South Africa; ^2^Department of Medical Biosciences, Faculty of Natural Sciences, University of the Western Cape, Cape Town, South Africa

**Keywords:** gold nanoparticles (AuNPs), proton therapy, radiation therapy, radiosensitization effect, dose enhancement effects, particle therapy, nanomedicine

## Abstract

The number of proton therapy facilities and the clinical usage of high energy proton beams for cancer treatment has substantially increased over the last decade. This is mainly due to the superior dose distribution of proton beams resulting in a reduction of side effects and a lower integral dose compared to conventional X-ray radiotherapy. More recently, the usage of metallic nanoparticles as radiosensitizers to enhance radiotherapy is receiving growing attention. While this strategy was originally intended for X-ray radiotherapy, there is currently a small number of experimental studies indicating promising results for proton therapy. However, most of these studies used low proton energies, which are less applicable to clinical practice; and very small gold nanoparticles (AuNPs). Therefore, this proof of principle study evaluates the radiosensitization effect of larger AuNPs in combination with a 200 MeV proton beam. CHO-K1 cells were exposed to a concentration of 10 μg/ml of 50 nm AuNPs for 4 hours before irradiation with a clinical proton beam at NRF iThemba LABS. AuNP internalization was confirmed by inductively coupled mass spectrometry and transmission electron microscopy, showing a random distribution of AuNPs throughout the cytoplasm of the cells and even some close localization to the nuclear membrane. The combined exposure to AuNPs and protons resulted in an increase in cell killing, which was 27.1% at 2 Gy and 43.8% at 6 Gy, compared to proton irradiation alone, illustrating the radiosensitizing potential of AuNPs. Additionally, cells were irradiated at different positions along the proton depth-dose curve to investigate the LET-dependence of AuNP radiosensitization. An increase in cytogenetic damage was observed at all depths for the combined treatment compared to protons alone, but no incremental increase with LET could be determined. In conclusion, this study confirms the potential of 50 nm AuNPs to increase the therapeutic efficacy of proton therapy.

## Introduction

Approximately 50% of the patients with malignant tumors receive radiotherapy (RT) as part of their initial cancer treatment (1). However, delivering a curative radiation dose to the tumor while limiting the dose to surrounding healthy tissue, remains one of the biggest challenges in RT. Furthermore, the physical location of the lesion may prevent effective and complete irradiation of the tumor. Despite recent advances in treatment planning and image-guided intensity-modulated RT, several new treatment strategies are continuously being developed (2). Particle therapy and novel radiosensitizers are part of these recent developments, which offer the potential to augment the therapeutic efficacy (2–5).

Gold nanoparticles (AuNPs) with a diameter of 100 nm or less, have several properties that make them ideal radiosensitizers, including their high atomic number (Z = 79), biocompatibility and low cytotoxicity (6, 7). Several preclinical studies illustrated that AuNPs are potent radiosensitizing agents (8–11). Most studies focused on conventional RT with high-energy megavoltage (MV) and low energy kilovoltage (kV) X-rays, as reviewed in (4, 9, 11–14). Up until now, the radiosensitizing effect of AuNPs are most pronounced for kV X-rays and while there is a motivation to use this radiation quality in the clinic alongside MV X-rays, its usage remains limited due to its shallow penetration depth in the patient (12, 15).

The application of AuNPs as potential radiosensitizers in particle therapy has recently gained momentum, reflected by an increase in both simulation and experimental radiobiology studies (16–18). The growing interest in this type of studies is closely linked to the emerging number of proton therapy (PT) facilities around the world, where the interplay with nanomedicine could potentially further improve the treatment outcome and enlarge the clinical scope. The rationale for the clinical use of proton beams is primarily motivated by their dosimetric advantage compared to conventional X-ray RT. In contrast to X-rays which are characterized by a depth-dose profile reaching a maximum after a short build-up of a few centimeters with an exponential attenuation thereafter, protons have a depth-dose profile with a low entrance plateau region that reaches a maximum peak just before the end of the proton range. This results in a depth dose curve with a sharp dose fall-off towards the end, beyond which no radiation dose is deposited. The range of protons depends on their initial energy and can be adjusted to treat tumors at different depths (19). By combining several proton beams of different energies, a spread-out Bragg Peak (SOBP) can be obtained to cover the target volume. This allows the positioning of the region of maximal energy within the treatment target, while limiting damage to surrounding healthy organs and tissues (20, 21). Due to the superior targeting, PT is arguably most beneficial for the treatment of tumors in proximity to critical organs at risk and for specific subsets of the population who are more prone to develop late effects, such as pediatric patients (22, 23).

The high-energy proton beams (60–260 MeV) that are used in clinical practice and MV X-rays are both considered to be low linear energy transfer (LET) radiation qualities. However, the energy of the protons drops rapidly at the end of their range, resulting in a higher ionization density and a corresponding increase in LET (24). This translates into more complex, unrepairable biological damage and an associated increase in the relative biological effectiveness (RBE) in the distal region of the SOBP (25, 26). However, despite evidence in support of a variable RBE for clinical proton beams, a fixed RBE of 1.1 is still adopted in clinical practice (27). This RBE of 1.1 indicates that the biological effectiveness of high-energy protons to kill tumor cells is only 10% higher than that of sparsely ionizing X-rays. From this point of view, protons are less attractive for the treatment of radioresistant tumors where heavier ions (such as carbon ions), with a higher RBE, are proven to be up to four times more effective than X-rays (28–30). Therefore, radiosensitizers, such as AuNPs, are a promising approach to amplify the proton dose that is delivered within the tumor tissue. Furthermore, the addition of AuNPs may decrease the heterogeneity in tumor response, which is caused by areas in the tumor microenvironment containing cancer stem cells and regions of hypoxia.

At first, AuNPs were not expected to be effective radiosensitizers in PT. Mainly due to the decrease in collision stopping power of charged particles as a function of Z, in contrast to the high photoelectric absorption with strong Z-dependence of kV X-rays. However, charged particles are nevertheless able to activate a non-linear avalanche of electron emissions from AuNPs and surface plasmon excitations can result in a large production of secondary electrons, which could also make AuNPs effective radiosensitizers in PT (18). A growing number of studies indicate that the Coulomb nanoradiator (CNR) effect and the chemical damage by reactive species plays a major role in the dose enhancement effects that are observed for high Z nanoparticles and high-energy proton beams (31, 32). The first biological assessments confirm the radiosensitization potential of AuNPs in PT, but this line of research is only at its beginning. The underlying mechanisms that are responsible for the observed radiosensitization effects are not completely understood and there are currently only a limited number of *in vitro* and *in vivo* studies combining proton irradiation and AuNPs (33–43). This *in vitro* study with larger 50 nm AuNPs was designed as a proof of principle to investigate the uptake, cytotoxicity, radiosensitization effect and the potential LET-dependence of this effect, for a high-energy (200 MeV) clinical proton beam.

## Materials and Methods

### AuNPs

Spherical AuNPs of 50 nm stabilized in a citrate buffer (Sigma-Aldrich Co. LLC, St. Louis, Missouri, United States) were stored at 4°C to ensure stability over time and filtered through 0.2 μm filters (Whatman, Maidstone, UK) before addition to the cells to ensure sterility. The size and stability of the AuNPs in suspension was confirmed using Ultraviolet-visible (UV-visible) spectroscopy, as previously described (44). AuNP colloidal solutions were recorded as a function of wavelength using a POLARstar® Omega (BMG Labtech, Ortenberg, Germany) UV-vis spectrophotometer from 400–800 nm at a path correlation of 2.94 and resolution of 1 mm. More details and results on AuNP characterization can be found in the Supplementary Material.

### Cell Culture

CHO-K1 cells were kindly donated by the Medical University of Southern Africa (passage 16) and originally purchased from the American Type Culture Collection (ATCC) (Manassas, Virginia, USA). This cell line was originally derived as a subclone from the parental CHO cell line initiated from a biopsy of an ovary of an adult Chinese hamster by T. T. Puck in 1957 (45, 46). Since this is a proof of principle study, this CHO-K1 cell line was selected as it is often used in radiobiology studies and its radiosensitivity was well characterized in previous studies at our institute (47, 48). Cells were cultured in RPMI-1640 medium [(Gibco, Dun Laoghaire, Dublin, Ireland) supplemented with 10% Fetal Bovine Serum (FBS) (Gibco) and 1% Penicillin and Streptomycin (Gibco)]. Incubation took place under standard cell culture conditions at 37°C in a humidified 5% CO_2_ atmosphere. The CHO-K1 cells were periodically screened for Mycoplasma.

### AuNP Uptake

To determine the quantity of AuNPs internalized by the cells, inductively coupled mass spectrometry (ICP-MS) (7900 ICP-MS Agilent, California, USA) was performed at the Central Analytic Facility (CAF) of Stellenbosch University. CHO-K1 cells were exposed to 2.5, 5 and 10 μg/ml of 50 nm AuNPs and incubated for 4 hours to mimic the exposure conditions of the proton irradiation experiments. The CHO-K1 cells were then harvested, counted, and exposed to aqua reagia (1:1 HNO3, HCL) to dissolve the AuNPs. The quantity of gold atoms in solution was detected in parts per billion (ppb) and subsequently converted to a volume (pg/ml) normalized for the counted cell number. Based on the outcome of these first uptake experiments, all consequent experiments were performed with the highest concentration of 10 μg/ml or 37 μM AuNPs for 4 hours (unless stated otherwise).

Transmission electron microscopy (TEM) was performed to visually confirm the presence of AuNPs within the CHO-K1 cells. As described above, the adherent cells were treated with 50 nm AuNPs and incubated. Cells exposed to the AuNPs were fixed in 4% paraformaldehyde and then placed in a series of heavy metal stains as described in (49, 50). Sections were visualized with a Zeiss MERLIN Field Emission Scanning Electron Microscope (FESEM) (Carl Zeiss, Oberkochen, Germany) operated at 6-8 kV acceleration voltage with a 10 nA probe current, using Backscattered Electron Detection. Electron images were captured as TIFF files, using a pixel averaging noise reduction algorithm.

### Cell Proliferation

The crystal violet assay was used to investigate the impact of AuNPs on the cell proliferation of CHO-K1 cells in the absence of proton irradiation. The difference in absorbance (λ_max_) between crystal violet (570 nm) and 50 nm AuNPs is about 10-60 nm, so spectral overlap can be excluded ensuring that false negative/positive results are prevented. The cells were seeded into three 96-well plates (Sigma Aldrich) at a density of 2,500 cells/well (population doubling time of this cell line is less than ±18 hours), allowed to adhere overnight, enter log phase, and treated with 10 μg/ml AuNPs for 4 and 24 hours. Cell cultures without AuNP treatment were incorporated in the experiment to serve as controls. Following the respective incubation periods, the cells were stained according to the methods described in (51). Briefly, cells were fixed in 1% Gluteraldehyde (Sigma), washed with Phosphate Buffered Saline (PBS), and stained with 0.5% Crystal Violet for 30 minutes. Thereafter, the plates were rinsed with dH_2_O and after drying overnight, 0.1% Triton-X 100 was used to solubilize the crystal violet and lyse the cells to extract proteins and other cellular organelles. The plates were at 570 nm using a POLARstar® Omega UV-vis spectrophotometer (BMG Labtech, Ortenberg, Germany) and the optical densities (OD_570_) recorded for each well. The average OD_570_ of the non-treated control cells at 4 and 24 hours was set to 100% to determine the percentage of viable, proliferating cells after exposure to AuNPs at the same time points.

### Proton Irradiation

**Figure 1:**
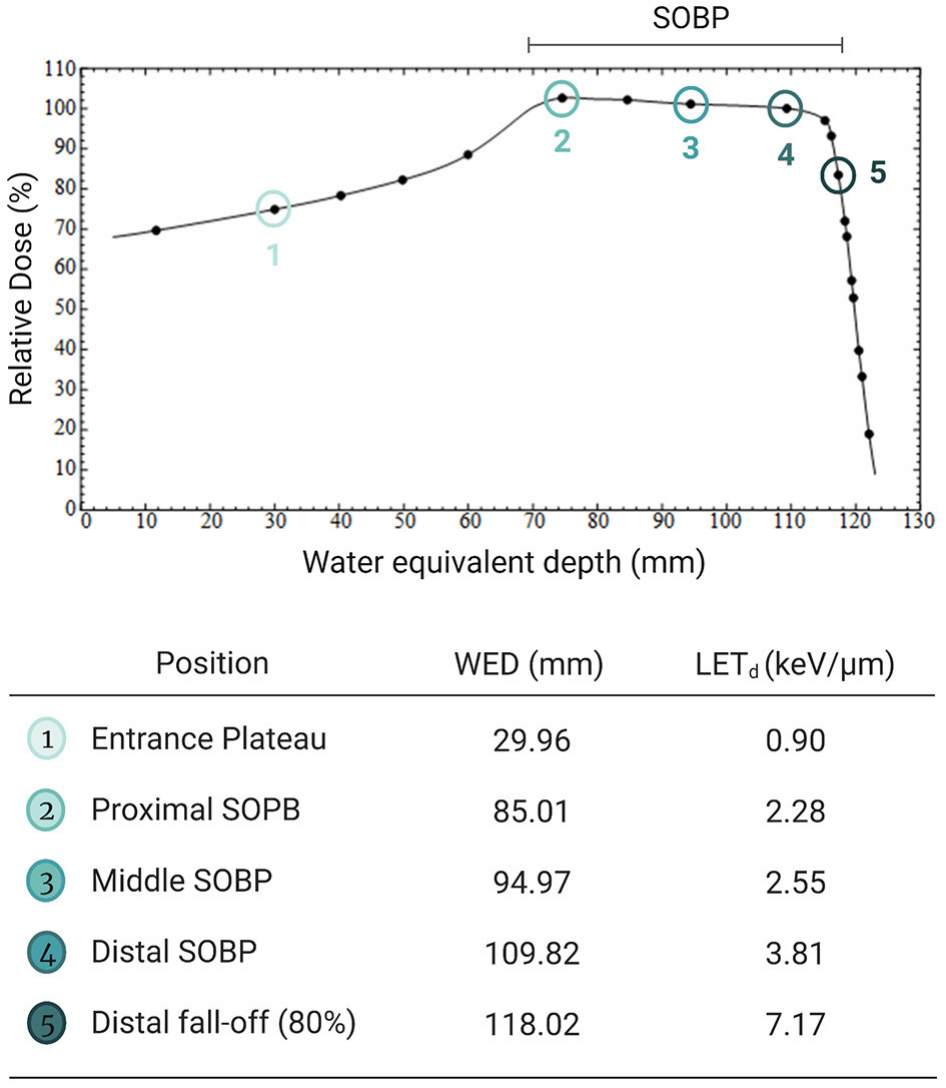
Illustration of the five different irradiation positions with a modulated 200 MeV proton beam with a 50 mm spread-out Bragg peak (SOBP) and a range of 120 mm; with the corresponding Water Equivalent Depth (WED) in the Perspex phantom and the increasing LET_d_ values. Created with Biorender.com.

The irradiations were performed with the 200 MeV passive scattering clinical proton beam line at NRF-iThemba LABS. For these experiments, the 200 MeV proton beam coming from the Separated Sector Cyclotron (SSC) was degraded to a modulated proton beam with a 50 mm SOBP, R50 range in water of 120 mm and a circular field size of 100 mm diameter was used (with an incident energy of roughly 120 MeV). All cell irradiations were performed in a Perspex phantom consisting of individual plates of various thicknesses which were placed upstream of the cells to obtain measurement positions at different water equivalent depths (WED) with increasing dose averaged LET (LET_d_) values as previously measured in (48). The physical depth-dose profile of the proton beam was measured with a Markus^TM^ ionization chamber (model 30-329) to determine the output factors (Gy/MU) at the different positions that were used for the cell irradiations (Figure 1). A monolayer of CHO-K1 cells was irradiated in a T25 cell culture flask (NEST Biotechnology Co., Ltd., Wuxi, China) perpendicular to the beam direction. For each assay, two sets of culture flasks containing CHO-K1 cells were irradiated, one with, and one without AuNPs exposure prior to irradiation. The media of all the culture flasks was replaced with new media just before irradiation, to ensure that only the AuNPs that were taken up by the cells would be responsible for the observed effects.

### Colony Survival Assay

CHO-K1 cells were seeded at a density 750 000 cells in T-25 flasks and allowed to attach overnight. Half of the cell culture flasks were treated with AuNPs, while the other half was left untreated. Following the incubation period of 4 hours, cells were harvested, counted, and seeded in triplicate into 60 mm petri-dishes (Greiner Bio-one, Kremsmunster, Germany). This ensured that cells could internalize AuNPs for the allotted incubation period prior to irradiations and that only effects of internalized AuNPs were considered as AuNPs were not left to react in media during irradiation. The seeding of cells whether pre- or post-irradiation have been shown to have negligible effects on cell behavior and data output (52). The petri-dishes were irradiated in the middle of the SOBP (Figure 1) with doses ranging from 2 to 8 Gy to produce a full dose response curve. After irradiation, the cells were placed at 37°C in a humidified 5% CO2 atmosphere to proliferate into colonies for 6 days (≥ 50 cells per colony), followed by fixation and staining (0.01% amido black). The number of visible colonies were then manually scored, where each colony is considered to represent a surviving cell. Firstly, the plating efficiency (PE) (with and without AuNPs) as described in (53) and denoted as equation 1 was considered:

(1) $$\begin{array}{r} PE=\;\frac{number\;of\;colonies\;formed}{number\;of\;cells\;seeded}\;\times \;100\% \end{array}$$

In this study, there was a PE_AuNP_ and a PE_control_. Thereafter, the surviving fraction (SF) was calculated for the different exposure conditions according to equation 2.

(2) $$\begin{array}{r} SF=\;\frac{number\;of\;colonies\;formed}{number\;of\;cells\;seeded\;\times PE}\;\times 100\% \end{array}$$

Experimentally obtained colony survival data was fitted using the linear quadratic (LQ) model, represented in equation 3.

(3) $$\begin{array}{r} S=\;e^{-\left(\alpha D-\beta D^{2}\right)} \end{array}$$

*S* represents the fraction of surviving cells for a dose (*D*) expressed in Gray (Gy), and α and β are the model constants.

To assess the radiosensitization effect of 50 nm AuNPs on proton irradiation, sensitization enhancement ratio (SER) was calculated as outlined in equation 4.

(4) $$\begin{array}{r} SER=\frac{Survival\;fraction\;without\;AuNPs\;\left(Control\right)}{Survival\;fraction\;treated\;with\;AuNPs\;\left(AuNP\;Treated\right)} \end{array}$$

Additionally, the amplification factor (AF) was calculated at different radiation doses ranging from 2 up to 8 Gy, to evaluate the amplification of radiation induced cell death. AF was calculated from the fitted surviving curve as follows (equation 5).

(5) $$\begin{array}{r} AF=\;\frac{\;SF^{\begin{array}{c} fitted\;curve\;\\ control\; \end{array}}-\;SF^{\begin{array}{c} fitted\;curve\;\\ AuNPs \end{array}}}{SF^{\begin{array}{c} Fitted\;curve\\ \;control \end{array}}}\;\times \;100\% \end{array}$$

### Cytokinesis-Block Micronucleus Assay

The cytokinesis-block micronucleus (CBMN) assay was used for scoring micronuclei (MNi), reflecting chromosome breakage or whole chromosome loss, because it is restricted to binucleated cells (BN) that have undergone one cycle of cell division. This prevents confounding effects caused by suboptimal or altered cell division kinetics (54). The CHO-K1 cells were seeded (750 000 cells/T-25 flask) and allowed to attach overnight, followed by treatment with AuNPs. Thereafter, the cells were irradiated at different positions along the SOBP (Figure 1) with a radiation dose of 2 Gy. Sham-irradiated control flasks were included. Immediately after irradiation, cytochalasin-B (2.25 ug/ml) was added to the flasks. The cells were incubated for 24 hours, trypsinized and centrifuged at 1000 rpms for 8 minutes and Permeabilized with Potassium Chloride (KCl). Afterwards, the cells were fixed in 10:1:11 Methanol/Acetic Acid/Ringer solution overnight. The next day, the cell suspension was centrifugated and further fixed with 10:1 Methanol/Acetic Acid solution. After fixation, 30 μl of the fixed cell suspension was dropped onto a glass slide according and stained in a 1% Acridine Orange solution, followed by 0.1 M Gürr Buffer and covered with a coverslip. MNi were scored manually with a 20 × objective in approximately 500 binucleated cells per slide using an Axioscope fluorescent microscope (Carl Zeiss). At least three slides were scored per condition and the average values were calculated. The assay was performed in biological triplicate.

### Statistical Analysis

All statistical analysis was performed using GraphPad Prism (version 5.0). Statistical comparisons were performed by way of paired t-test and/or one-way analysis of variance (ANOVA). A significance level (α) of 0.05 was used in all tests, hence *P* < 0.05 were considered statistically significant, *p* < 0.01 highly significant and *p* < 0.001 extremely significant. Data is reported as average values ± standard deviation (SD). All assays were performed in biological triplicate.

## Results

### AuNP Uptake

**Figure 2:**
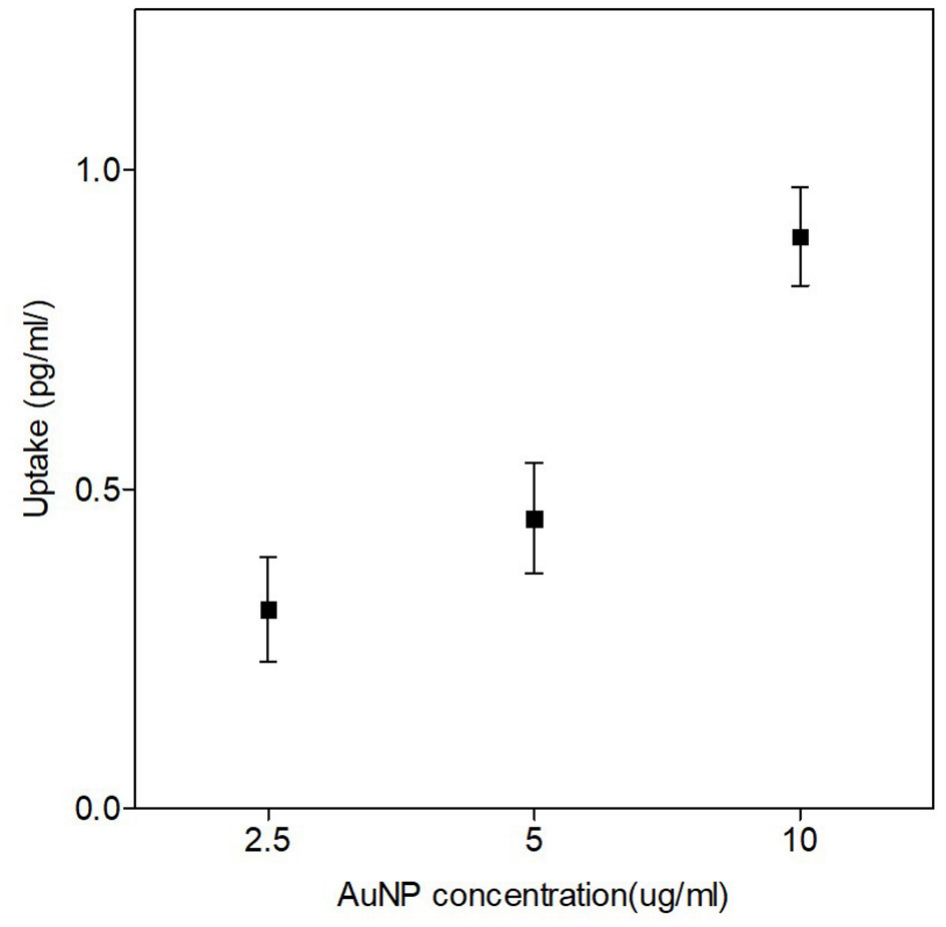
Quantities of AuNP internalized per cell after a 4-hour incubation with different concentrations of 50 nm AuNPs. ICP-MS results show the dose-dependent uptake of AuNPs. The highest internalization of 50 nm AuNPs was observed at a concentration of 10 μg/ml (37 μM). The error bars represent the standard deviation of three biological replicates per concentration.

**Figure 3:**
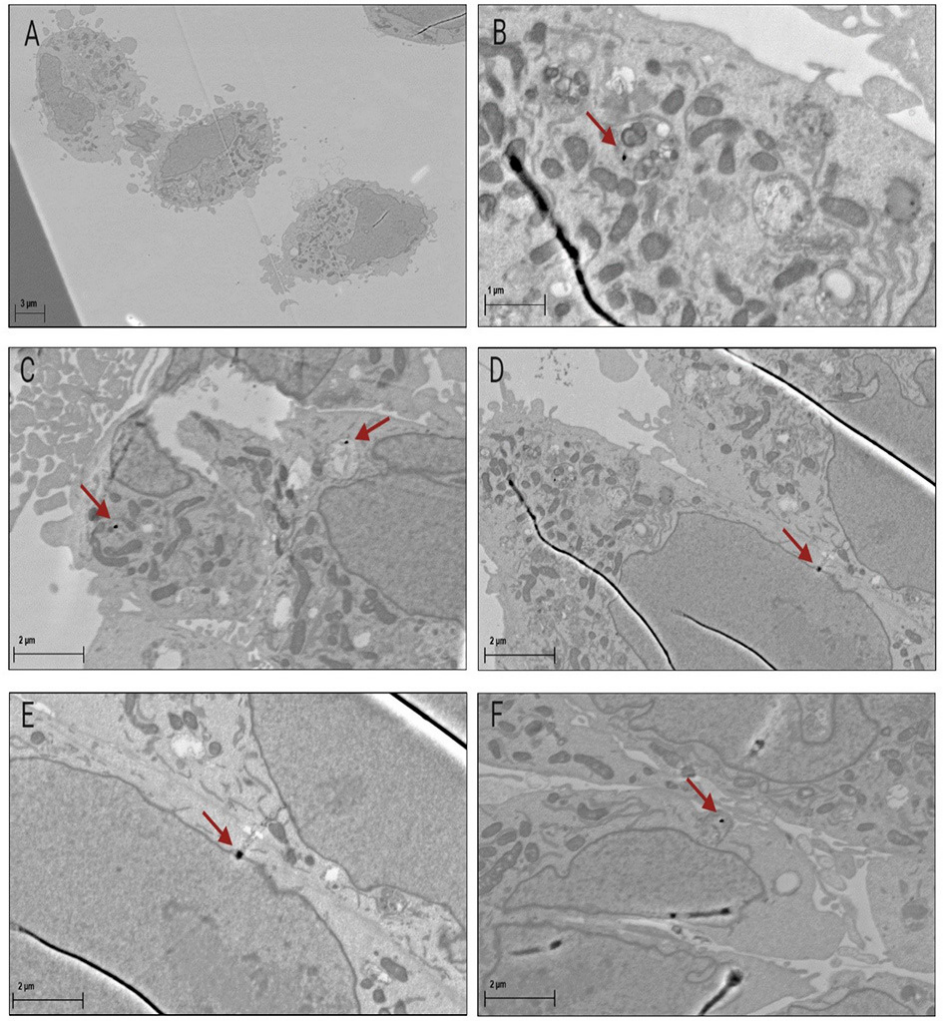
Confirmed uptake of AuNPs in CHO-K1 cells with TEM. Image **(A)** represents three untreated control cells captured at a higher magnification than panels **(B-F)**, which represent cells that were exposed to 50 nm AuNPs. Images **(B–F)** show that low numbers of AuNPs were taken up by the cells, but were successfully internalized. AuNPs localized randomly into vacuoles within the cells **(B, C** and **D)**. AuNPs were also located within proximity to the nuclear membrane **(F)** as well as integrated into the nuclear membrane of the cells **(D** and **E)**.

The uptake and localization of the 50 nm AuNPs in CHO-K1 cells was confirmed with ICP-MS and TEM respectively. For the ICP-MS experiments, the cells were exposed to different low concentrations of 50 nm AuNPs (2.5, 5 and 10 μg/ml) for 4 hours, based on concentrations and incubation times that were used in previous studies (36, 55). A dose dependent uptake in the CHO-K1 cells was observed, with an increase from 0.31 ± 0.047 pg/ml for the lowest concentration up to 0.89 ± 0.044 pg/ml of gold per cell for the highest concentration (Figure 2). The gold uptake per cell increased by almost a factor 3 and therefore, it was decided to select the highest concentration of 10 μg/ml (37 μM) for the proton irradiation experiments. Additionally, TEM provided visual confirmation of AuNP internalization and showed a random distribution of AuNPs throughout the cytoplasm of the cells, with some close localization to the nuclear membrane but no nuclear entry was observed (Figures 3A–F).

### Impact of AuNPs on Cell Proliferation

To determine the impact of the 50 nm AuNPs on the viability of the CHO-K1 cells, cell proliferation was assessed with a crystal violet assay at two incubation times of 4 and 24 hours. A minimal impact on cell proliferation was observed in the cultures that were exposed to 10 μg/ml AuNPs compared to the non-treated cultures at both time points (4 and 24 hours), with a non-statistically significant decrease to 89.45 ± 13.87% and 93.87 ± 8.2% in the exposed cultures respectively.

### Radiosensitization Effect of AuNPs Evaluated With the Colony Survival Assay

**Figure 4:**
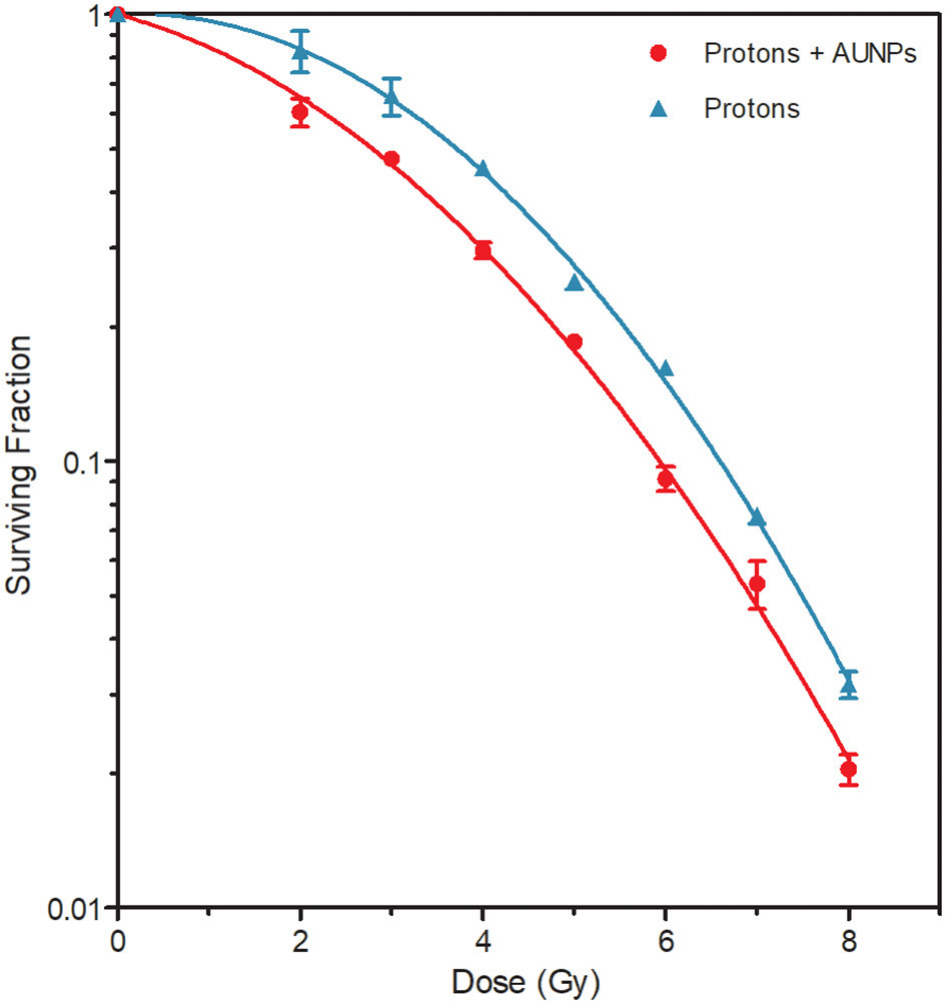
Surviving fractions of cells after the concurrent treatment with various doses of protons, with (red) and without (blue) internalized 50 nm AuNPs. All samples were irradiated in the middle position of the SOBP. The values represent the average SF and standard deviation of three biological repeats.

The combined effect of AuNPs and protons on cell killing was investigated by the colony survival assay. A paired comparison revealed a statistically significant reduction in cell survival was observed between the cells that were pre-treated with AuNPs and irradiated with protons, compared to the cells that were irradiated with protons alone (Figure 4) (*p* < 0.05). By fitting the linear quadratic model to the cell survival fractions, α-values of 0.023 ± 0.017 and 0.125 ± 0.019 and β-values of 0.056 ± 0.002 and 0.044 ± 0.003 were obtained for protons alone and protons combined with AuNPs respectively. The sensitization enhancement ratio (SER) was calculated at 10 and 50% survival as described in (35, 39), resulting in a SER values of 1.11 and 1.33 respectively. These results confirm the radiosensitization effect of 50 nm AuNPs, which resulted in an increased cell killing effect with proton irradiation. Furthermore, the amplification factor (AF) was calculated for the different radiation doses used in this study, as previously described in (35, 37). The largest AF of 43.8% was observed at a proton dose of 6 Gy, while the AF at a clinically relevant fractionation dose of 2 Gy was 27.1%.

### Evaluation of the LET-Dependence of AuNP Radiosensitization Using the CBMN Assay

**Figure 5:**
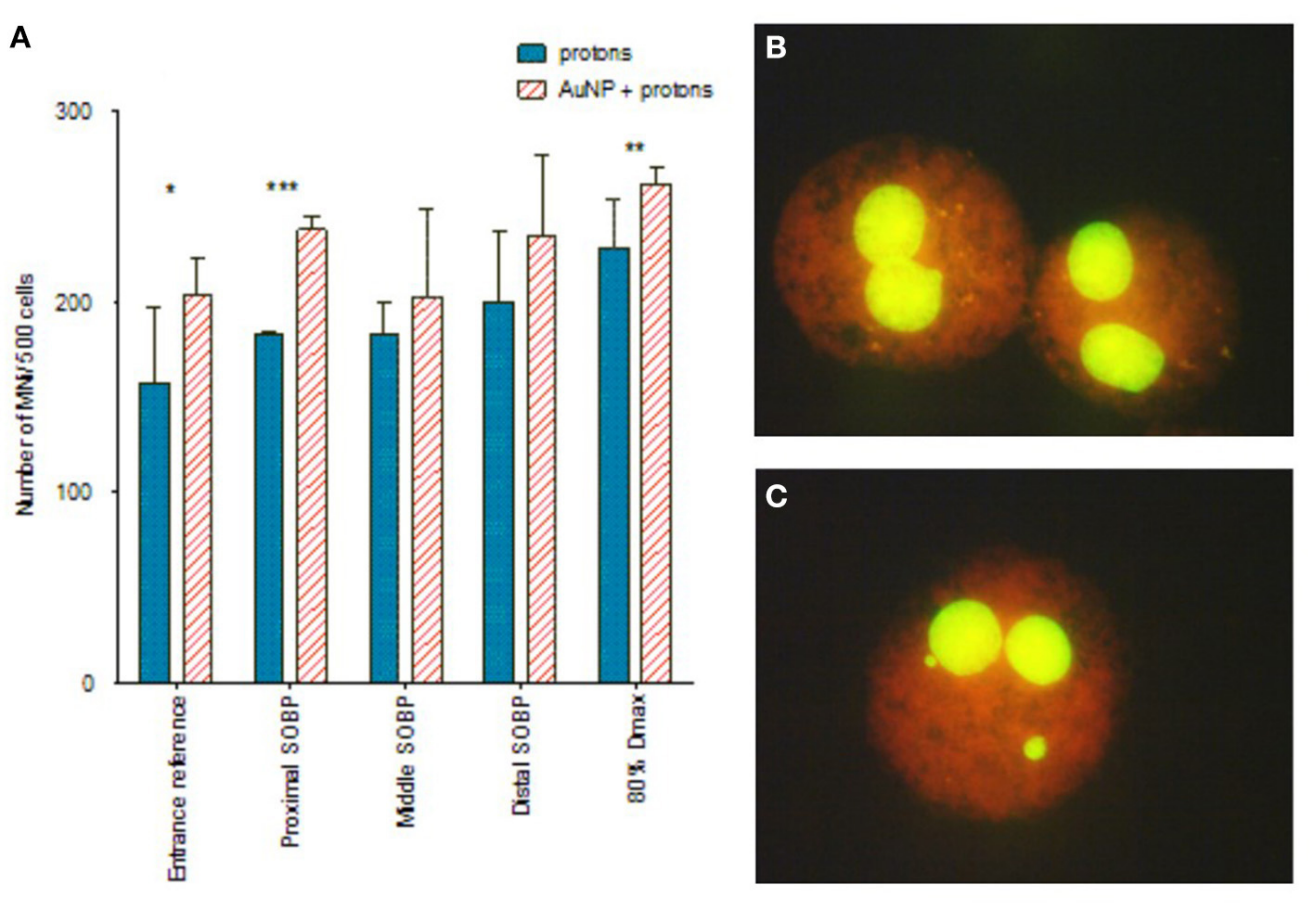
**(A)** The effect of protons and AuNPs on the MNi expression of CHO-K1 cells. Where (*) denotes *p* < 0.05, (**) = *p* < 0.01 and (***) = *p* < 0.001. **(B)** Binucleated cells without MNi (proton alone). **(C)** Binucleated cells containing MNi (protons + 50nm AuNPs). The plotted values in the graph represent the average number of MNi/500 BN cells and the respective standard deviation of three biological repeats. Created with Biorender.com.

To explore whether the radiosensitization effect of AuNPs is dependent on the LET of the proton beam, the CBMN assay was performed at five different positions along the SOBP. The CBMN assay was selected over the colony survival assay for this evaluation, since it has a higher sensitivity to detect slight changes in the radiosensitization effect. Induced MNi frequencies are reported for this comparison, which means that the average background MNi values were deducted from the values obtained with proton irradiation. These values were 13.00 ± 2.61 MNi/500 BN cells and 15.50 ± 6.47 MNi/500 BN cells for the unirradiated samples without AuNP incubation and with AuNP incubation respectively. There was no statistically significant difference between both non-irradiated control values. This confirms that the 4 hours incubation with 50 nm AuNPs does not induce a cytotoxic effect on the CHO-K1 cells, which is in line with the cell proliferation results. As expected, an increase in chromosomal damage was observed with increasing SOBP depth (or LET) in cells exposed to 2 Gy proton irradiation in the absence of AuNPs (Figure 5). Using the entrance plateau position as a reference, the MN frequency showed a gradual increase with a factor of 1.16 ± 0.30, 1.16 ± 0.11, 1.27 ± 0.26 at the proximal, middle and distal end of the SOBP; going up to 1.45 ± 0.32 at the distal fall-off position. This confirms the expected increase in DNA damage and RBE at the end of the proton range.

Paired analysis showed that pre-incubation with AuNPs significantly enhanced the chromosomal damage at all positions along the SOBP when compared to the results obtained with proton irradiation alone (*p* < 0.01). This finding supports the radiosensitization effect of AuNPs observed with the colony survival assay, but one should consider that the error bars on the average MNi frequency are large at some positions. A statistical analysis of the MNi results per individual position only shows a statistically significant radiosensitization effect of AuNPs at the entrance plateau, proximal SOBP and 80% D_max_ position (Figure 5). In addition, no incremental increase with LET was observed for the combined treatment with AuNPs, so this proof-of-principle study does not illustrate a potential LET-dependence of the radiosensitization effect.

## Discussion

The combination of the excellent sparing of surrounding healthy tissue with PT and the potential of AuNPs to enhance the biological effect within the tumor, could offer a new opportunity to increase the clinical efficacy of PT. While many questions remain unsolved, the initial biological findings are encouraging and boost future research efforts on the synergistic effects of PT and AuNPs (16, 17). Since the pioneering experiment of Hainfeld et al., the number of biological studies using kV and MV X-rays are steadily growing (5, 9, 11, 12, 56–61). The number of experimental studies that investigated the radiosensitization effect of AuNPs in PT are currently still limited, and are summarized in Table 1 (33–43). However, as outlined in Table 1, the PT studies show considerable differences in experimental conditions including variations in AuNP size, shape, and functionalization as well as exposure conditions such as incubation times, concentration, and proton beam energy. This underlies the differences in experimental findings and consequently impedes conclusions on the potential of AuNP radiosensitization in PT.

**Table 1:** Overview of the existing radiobiological studies which investigated the potential radiosensitization effects of AuNPs in combination with proton irradiation.

**References**	**Incoming proton beam energy (radiation quality)**	**Gold nanoparticle size**	**Concentration**	**Incubation time**
Polf et al. (33)	160 MeV (within SOBP region)	±44 nm AuNP phage nanoscaffolds	1 ng/cell	Not defined
Kim et al. (43)	45 MeV (within SOBP region)	2 and 13 nm AuNP	0.1–2 mg/ml	Overnight
Kim et al. (34)	45 MeV (Bragg peak/entrance plateau)	5 nm ligand coated AuNPs	100 or 300mg/kg (*in vivo*)	1, 6, 12, 24 and 48 h
Penninckx et al. (35)	1,3 MeV (LET: 25 keV/μm)	10 nm conjugated AuNPs	50 ug/ml	6 and 24 h
Jeynes et al. (36)	3 MeV (LET: 12 keV/μm)	50 nm conjugated AuNPs	5,5 ug/ml	4 h
Li et al. (37)	<2 MeV (LET: 10 or 25 keV/μm)	5 and 10 nm amine functionalized AuNPs	0,05 mg/ml	24 h
Li et al. (38)	1,3 MeV (LET: 25 keV/μm)	±40 nm Cetuximab AuNPs	5 ug/ml	30 min
Abdul Rashid et al. (39)	150 MeV (within SOBP region)	1.9 nm AuNP nanoprobes	1 mMol/L	Not defined
Torrisi et al. (40)	2.0 MeV	5 nm AuNP	5.5 × 10 ^13^ NPs/ml	1 week
Enferadi et al. (42)	200 MeV (within SOBP region)	1.8 nm conjugated AuNP	90 μg (45μg/ml)	24 h
Liu et al. (41)	3.0 MeV	6.1 ± 1.9 nm coated AuNP	500 μM (41)	Not defined

The 4 hours incubation time in this study was based on the findings of Chithrani et al., a foundational report for many AuNP based experiments, where a significant uptake of 50 nm AuNPs was observed via suspected endocytosis in the first 2 hours, reaching a plateau after 4–6 hours (55). The same rationale was applied in the study of Jeynes et al. who also used 50 nm AuNPs (36). The relatively short incubation time was particularly helpful to counter potential delays in beam delivery, which are inherent to experiments at accelerator facilities. Previous studies showed that AuNP update and cytotoxicity are cell type dependent, with a preferential uptake by cancer cells in comparison to normal cells (62–65). This brings us to one of the main limitations of the current proof of principle study, since only one non-cancerous cell type was used for this evaluation.

However, the size of the AuNPs might have an even larger impact on the uptake than the cell type. Several studies reported maximum uptake and retention within the cells for 50 nm AuNPs (55, 63, 66, 67). The efficient suspected endocytic capabilities of the 50 nm AuNPs are conjectured to be due to the similarity in required vesicle size for the initial cellular entry of several viruses (68). In this context, it is worth to mention that the hafnium oxide nanoparticle NBTXR3 (Hensify®), which also has a size of 50 nm, is currently undergoing several clinical trials (NCT02721056; NCT02379845) and making its way to the clinic for combinations with RT as a radio-enhancer (69). It is anticipated that nanoparticles up to 100 nm in diameter enter the cells via clathrin-mediated endocytosis (70, 71). On the contrary, AuNPs smaller than 30 nm might leave the cell again by passive diffusion (72, 73). However, nanoparticle internalization can occur via a vast array of mechanisms (74, 75), and at present, definitive conclusions cannot be made regarding the precise mechanism of nanoparticle entry in this study, but it is most likely by endocytosis. As a proof of principle study on the potential radiosensitization effect of AuNPs in PT, uncoated, standard AuNPs were used in this work. TEM micrographs show AuNPs update in the cytoplasm of cells (Figure 3B) and some AuNP were even located close to the nuclear membrane (Figure 3D, E). However, the specific type of endocytosis that was responsible for the uptake in this study requires further investigation (76). Since several studies demonstrated that larger AuNPs exhibit lower *in vitro* cytotoxicity compared to smaller AuNPs (up to 5 nm), this provided an additional motivation to select AuNPs with a size of 50 nm (77–79). The cell proliferation results showed no significant *in vitro* cytotoxic effects in CHO-K1 cells after an incubation period of 4 hours. The low cytotoxicity is in line with previous observations for 50 nm AuNP sizes (77, 80). Even after 24 hours, there was only a minimal, non-significant decrease in cell proliferation observed in this study. However, it is also important to take into consideration that larger AuNPs will result in an increased self-absorption resulting in a loss of the desired dose enhancement effect (32). It is therefore important to look for the ideal balance between the gain in enhancement due to the greater gold mass and the self-absorption, which will also depend on how the AuNPs cluster within tumor cells and the incident proton energies. Furthermore, the charge of the AuNPs could also influence the result, as findings by Goodman et al. showed that positively charged AuNPs were cytotoxic whereas a later study by the same group showed no cytotoxicity with negatively charged AuNPs (81). The charge of the AuNPs in this study was negative (−35.1 mV), possibly protecting the cells against cytotoxicity.

Several simulation studies have investigated the potential dose enhancement effects of AuNPs in PT *in silico*. One of the first studies came from Walzlein et al., who explored the possible dose enhancement at nanoscale level with monoenergetic proton beams at energies of clinical interest (82). The study reported a relevant increase in local dose around the nanoparticle, which was mainly attributable to the production of low-energy electrons (including Auger cascades). The Auger cascades are limited to a very short nanometer range around the nanoparticle which limit the chance of interaction with the DNA. Even though the Auger electrons do not always reach the DNA, their effects are not negligible (83). A comprehensive overview of Monte Carlo studies on proton interaction with NPs can be found in (16, 17, 84). Alternative biological mechanisms for the observed AuNP radiosensitization have been hypothesized over the past few years, such as enhanced reactive oxygen species (ROS) production (12, 85). This biological, instead of physical enhancement effect has recently been supported by the *in-silico* findings of Fuss et al. (83) and Peukert et al. (86). Although physical effects are not entirely outside of the realm of possibilities, their dose enhancement effects are localized. It is therefore expected that biological pathways are more likely to play a key role in the observed effects. The results in this study are closest to the Monte Carlo study of Martinez-Rovira and Prezado where 4 and 50 nm AuNPs were irradiated with several proton beam configurations (87). While a dose enhancement of 1.7 was observed for the 50 nm AuNPs, the local dose enhancement effect was negligible when a more realistic beam configuration was used with the source further away from the target. Again, this illustrates that physical effects seem to play a minor role in the amplification of the biological effect and confirms that biological and chemical processes may be responsible for the enhanced radiosensitization in biological studies.

A statistically significant decrease in cell survival was observed between the CHO-K1 cells irradiated with protons in the absence of AuNPs and the irradiated cells containing AuNPs (Figure 4). This finding supports the radiosensitization effect described by Abdul Rashid and co-workers, in which an SER_50_ of 2.64 was reported (39). However, this is considerably higher than the SER_50_ of 1.33 in this study. The SER_10_ in this study was only 1.11, while the study of Enferadi et al. reported a very similar SER_10_ value of 1.17 for a high energy proton (200 MeV) beam, however, very small AuNPs (1.8 nm) and a murine glioma cell line was used for the colony survival analysis (42). The AF was also calculated in this study, which is an illustration of the enhance proportion of dead cells in cultures with and without AuNPs that have been exposed to proton irradiation. The AF value reported by Li et al. was approximately 30% at 2 Gy using 10 nm AuNPs is relativelyclose to the AF at 2 Gy of 27.1% for 50 nm AuNPs in our study, while Enferadi et al. report and AF at 2 Gy of 17.7% (37, 42). Enferadi et al. calculated their highest AF value of 70.4% at 6 Gy, while the AF value in our study was also highest at 6 Gy in our study, but only 43.8% (42). However, it is important to note that the differences in cell lines, incubation times and AuNP size, will result in cell uptake variations as well as observed radio-enhancement effects. In addition, the LET of the proton beam varies significantly, which contributes to the discrepancies in different *in vitro* studies. As outlined in Table 1, there is very little consistency in the methodology of the *in vitro* studies that are published so far.

As expected, proton irradiation induced an incremental increase in MNi frequency with increasing depth along the proton SOBP and reached a maximum at the distal fall-of position (Figure 5). This is a direct consequence of the increase in ionization density with depth along the SOBP, which is also reflected in the increasing LET values in Figure 1. When cells were exposed to both AuNPs and protons, greater levels of chromosomal damage were observed at all positions compared to proton irradiation in the absence of 50 nm AuNPs. This effect did not increase gradually with LET which contradicts the previous observations of Li et al., where a LET-dependent radiosensitization was observed between 5 and 10 nm AuNPs (37). However, findings in this study are in line with the recent study of Fuss et al., who reported a lower efficiency of AuNP radio-enhancement at low particle energies close to the track-end (83). To date, no complete explanation for the LET dependence is available. In the present study, the radiosensitization effect of the AuNPs on chromosomal damage is highest at the entrance plateau and proximal SOBP position (Figure 5), which confirms this hypothesis. Despite the fact that this study was only performed with one cell line and designed as a proof of principle study, it presents the first *in vitro* results on the potential LET dependence of the AuNP radiosensitization effect with a proton beam of therapeutically relevant energy. The LET values in the current study are similar to the LET values applied by Schlathölter et al. to investigate the nanoscale damage of 3 nm platinum (Z = 78) and 5 nm gadolinium (Z = 64) nanoparticles using plasmid DNA probes with a proton energy of 150 MeV (88). The LET values of 0.44 and 3.6 keV/μm were representing the radiation quality at the entrance and the end of the proton track respectively, which are close to the LET values used in the current study of 0.90 keV/μm at the entrance plateau and 2.28–3.81 keV/μm in the SOBP (Figure 1). The beam quality used in the current study is closer to clinical practice than the high LET values applied in studies with low-energy proton beams listed in Table 1. While low-energy proton beams can be used as a substitute of high-energy proton beams to study radiobiological effects in the distal fall-off region, it is important to take some differences into consideration. The momentum spread (or energy spread) of the incident beam from an accelerator increases with the beam energy and is therefore significantly larger for high-energy beams compared to low-energy beams. The straggling of the protons near the distal edge of the beam also increases significantly as the beam energy increases. As a result of these two factors, the distal fall-off of a high-energy proton beam is considerably wider compared to a low-energy beam. Furthermore, due to these two factors, the proton energy spectrum for a high-energy proton beam is expected to be broader at a given relative position in the distal falloff, resulting in a lower fluence-weighted LET for a high-energy proton beam compared to a low-energy beam (89). Additionally, the secondary radiation field of a low-energy proton beam ( ≤ 8 MeV) differs from a high-energy proton beam since inelastic nuclear scattering processes and non-elastic nuclear reaction channels are closed at these lower energies. It was decided to perform the colony survival experiments in the mid-SOBP position (position 3 in Figure 1). Due to the weighted superposition of proton beams to form a clinical SOBP, we consider this position with its corresponding LET to be a representative location to mimic tumor response.

While the radiation quality in this study is more applicable to clinical practice, it is paramount to note some limitations of the current proof-of-principle study. The use of untargeted AuNPs could be a limitation, however this “passive targeting” approach has been applied by other groups (90, 91). In clinical practice, this principle is based on the enhanced permeability and retention (EPR) effect, which is attributable to the leaky tumor vasculature and doesn't require a targeted delivery mechanism to accumulate AuNPs in the tumor. However, there are constraints to this approach, including arbitrary targeting and inefficient dispersion in the tumor. Additionally, not all tumors exhibit the EPR effect and the AuNP uptake seems to be cell type dependent, while only one cell line was used in this proof-of-principle study (63, 92). Therefore, active targeting by functionalizing the surface of AuNPs with suitable tumor specific ligands that have a specific affinity to interact with the tumor cells, might be a more advisable approach to obtain higher intra-tumoral concentrations of AuNPs *in vivo* (93). This is another limitation in the current study, since the TEM images show only a very low number of AuNPs which are localized in the cells. However, these AuNPs are freely distributed and not localized in endosomes. According to Lin et al., the AuNPs freely distributed in the cytoplasm can result in a higher dose enhancement than those aggregated inside the endosomes because of lower internal absorption of secondary electrons in the AuNPs (42, 94).Provisional *in silico* results show that AuNP shell coatings lead to a decreased electron yield, which may not be beneficial to the improvement of RT in the presence of AuNPs (85). A recent *in vitro* study of Klebowski et al. described the radiation enhancement effect of bimetallic gold-platinum nanocauliflowers, with a highly developed surface area and average size of 66 nm, for the treatment of colon cancer with PT (95). A clinical proton therapy system (IBA Proteus C-235 cyclotron) with a beam energy of 225 MeV was used for these experiments, which showed a significant reduction in cancer cell viability compared to normal cells. Another alternative approach is the application of iron oxide nanoparticles (FeO NPs) as radiosensitizers. Their systemic toxicity is lower than gold or carbon nanomaterials, since they are efficiently degraded to ferritin, which can be assimilated by the body (96). A previous study of Kim et al. showed an inferior radiosensitizing efficacy of FeO NPs compared to AuNPs in combination with protons. However, recent study with magnetosomes showed increased radiosensitization (43, 97). The radiosensitizing potential of magnetosomes was obtained with both X-ray and PT, both *in vitro* and *in vivo* (97). Unfortunately, the proton beam energy is not defined in the paper, but the description points to a clinical proton beam line (energy > 45 MeV).

In conclusion, this study confirms the radiosensitization potential of AuNPs in PT, which may enhance the therapeutic efficacy of PT as a cancer treatment modality. However, more biological studies are needed to confirm the LET independence that was observed in this study and to identify the underlying biological and chemical mechanisms that are responsible for the radiosensitization of larger (50 nm) AuNPs in PT. Finally, the lack of conformity amongst biological assessments makes it difficult to correctly compare findings from different groups. Future studies into this field require standardization, including more careful consideration of the selection of AuNP size, concentration and irradiation conditions.

## Data Availability Statement

The original contributions presented in the study are included in the article/Supplementary Material, further inquiries can be directed to the corresponding author/s.

## Author Contributions

CC, CV, MK, and JS conceptualized, designed the experiments, analysed the data, and performed the statistical analysis. ME, XM, CC, and CV performed the irradiation experiments, the optimization and execution of the laboratory work. CV, CC, and MK wrote the paper while all authors contributed to and approved the final version of the article. CV, MK, and JS were responsible for the funding of the study. All authors contributed to the article and approved the submitted version.

## Conflict of Interest

The authors declare that the research was conducted in the absence of any commercial or financial relationships that could be construed as a potential conflict of interest.

## Publisher's Note

All claims expressed in this article are solely those of the authors and do not necessarily represent those of their affiliated organizations, or those of the publisher, the editors and the reviewers. Any product that may be evaluated in this article, or claim that may be made by its manufacturer, is not guaranteed or endorsed by the publisher.
