# Supplementary material for: Radiosensitization Effect of Gold Nanoparticles in Proton Therapy
Source: Front Public Health. 2021 Jul 29;9:699822. doi: 10.3389/fpubh.2021.699822 (PMC8358148; doi:10.3389/fpubh.2021.699822)
Supplement: Supplementary file 1 [file Data_Sheet_1.docx]

Supplementary Material

# UV-VIS Spectroscopy

UV-vis spectra of the 50 nm AuNPs were recorded as a function of wavelength using a POLARstar® Omega UV-vis spectrophotometer (BMG Labtech, Ortenberg, Germany) at 400- 800 nm at a path correlation of 2.94 and resolution of 1 nm. Supplementary Figure 1 illustrates the spectral curve confirming the 50 nm AuNP stability and size conformity.

# Zeta Potential and Dynamic Light Scattering determination

To determine the hydrodynamic size, charge, and polydispersity index of 50 nm AuNPs, 1 ml of AuNPs in colloidal suspension were placed into cuvettes or capillary tubes for Dynamic Light Scattering (DLS) and Zeta Potential (ZP) measurements, respectively. Samples were analyzed using a Zetasizer Nano ZS (Malvern, Worcestershire, United Kingdom). Information was retrieved in the phase analysis light scattering mode at 25°C. Supplementary Figure 2 confirms the negative charge of the AuNPs and the hydrodynamic size of 65.54 d.nm.

# Stability testing

Prior to AuNPs interacting with living cells or organisms, their surfaces are confronted to biological fluids such as cell culture medium, lung fluid or blood, whose components will inevitably interact with the nanoparticle surface. It is important to precisely understand the behavior of the 50 nm AuNPs in biologically relevant surroundings. Based on a representative model system developed by Balog *et al.*, AuNPs were incubated in commonly used buffers and cell culture media for 24 hours reflecting the longest incubation period of the AuNPs within cultures during experimental exposure scenarios (39). AuNPs were incubated in four different increasingly biologically complex media i) Phosphate Buffer Saline (PBS), ii) Bovine Serum Albumin (a high molecular weight protein and major component of serum (5 mg/ml), iii) RPMI 1640 medium, and iv) RPMI 1640 medium supplemented with 10 % FBS at both 25°C and 37°C.

As illustrated in supplementary Figure 3, Media (RPMI 1460) and supplemented media affected the spectral shifts the most with upward shifts of 5 nm in the absorbed spectra for all samples. SPR (surface plasmon resonance) flattening occurs in media containing AuNPs when compared to absorbance profiles without biological media, indicating AuNP agglomeration within the solutions. The AuNP agglomeration levels also increased when exposed to incubation temperatures of 37 °C as opposed to 25 °C.

# Supplementary Figures


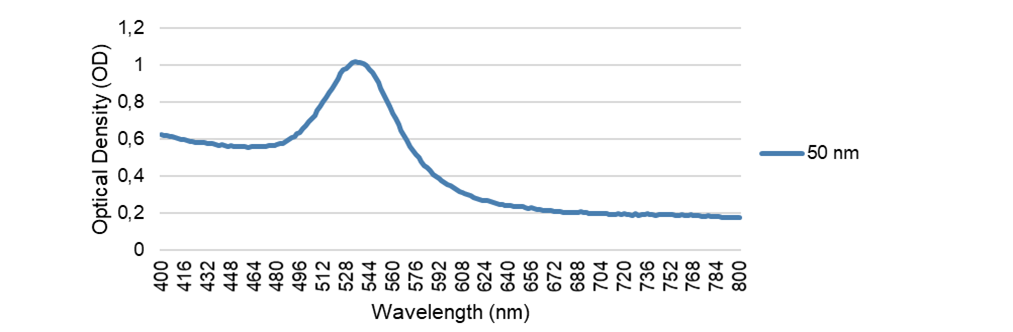


**Supplementary Figure 1.** Spectral curve confirming the 50 nm AuNP stability and size conformity (λ_max_: 546 nm)


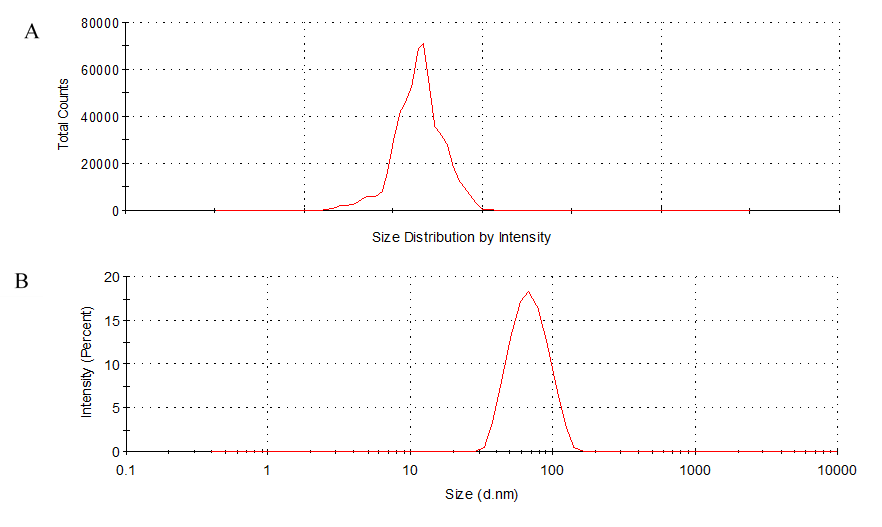


**Supplementary Figure 2.** A) Zeta potential analysis confirming the charge of AuNPs (-35.1mV). B) Dynamic Light Scattering exhibits the size the distribution of AuNPs confirming its hydrodynamic size (65.54 d.nm).


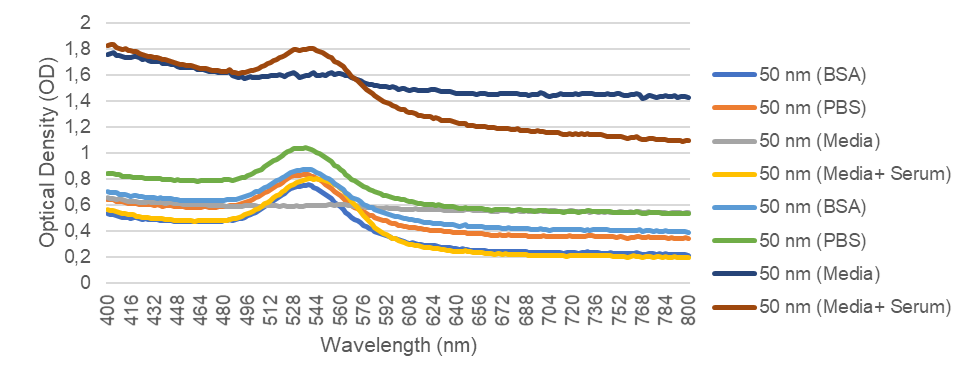


**Supplementary Figure 3.** Spectral curves of AuNPs after 24-hour incubation in bioreagents at 25oC (top panel) and 37oC (Bottom panel) respectively.
